# Malnutrition-induced frailty and risk of early rehospitalization in heart failure: A Survival analysis from a prospective cohort study

**DOI:** 10.12669/pjms.42.7.13245

**Published:** 2026-07

**Authors:** Farhan Muhammad Qureshi, Nazeer Khan, Riffat Sultana

**Affiliations:** 1 Farhan Muhammad Qureshi, MS-Public Health & Health Promotion, PhD Professor, Department of Community Medicine & Public Health Karachi Institute of Medical Sciences, Malir Cantt. National University of Medical Sciences (NUMS) Baqai Medical University, Karachi, Pakistan; 2 Nazeer Khan, PhD. Director ORIC (Office of Research, Innovation & Commercialization), Baqai Medical University, Karachi, Pakistan; 3 Riffat Sultana, FCPS, FACC, MD Consultant Cardiologist, Karachi Institute of Heart Diseases, Pakistan

**Keywords:** Consecutive Sampling, Cox regression, Frailty, Malnutrition, Nutritional frailty, Heart Failure, Hospital readmission, Kaplan-Meier plot, Rehospitalization, Survival analysis

## Abstract

**Objective:**

To evaluate the impact of malnutrition-induced frailty on time to rehospitalization among patients with heart failure (HF) in a tertiary care setting in Pakistan.

**Methodology:**

A prospective cohort study of three hundred participants was conducted at the Karachi Institute of Heart Diseases from November 2024 to April 2025, using non-probability consecutive sampling. Adults aged >40 years with confirmed HF and on a regular oral diet were enrolled at index admission and followed for 90 days. Malnutrition was assessed using the Malnutrition Universal Screening Tool (MUST; score ≥1) and frailty using the Tilburg Frailty Indicator (TFI; score >5). Patients with both conditions comprised the exposed group; those without either formed the unexposed group. Time to first rehospitalization was the primary outcome. Kaplan-Meier analysis compared survival curves, and Cox proportional hazards regression identified independent predictors of rehospitalization.

**Results:**

Kaplan-Meier analysis showed significantly shorter rehospitalization-free survival in the exposed group (log-rank p = 0.008). Although frailty was associated with a higher hazard of rehospitalization (HR = 4.912; 95% CI: 0.752-32.071), it did not reach statistical significance (p = 0.096) in the multivariable Cox model. BMI emerged as the only significant predictor (p = 0.047), with underweight patients at greater risk. No significant interaction was observed between BMI and frailty.

**Conclusion:**

Malnutrition-induced frailty showed a trend towards shorter time to rehospitalization in HF patients, though this association was not found statistically significant. Routine frailty screening and targeted post-discharge interventions may help reduce preventable readmissions, particularly in resource-limited settings.

## INTRODUCTION

Unplanned rehospitalizations following discharge from an index admission are widely recognized as benchmarks of healthcare quality.^1^ However, they also pose a global challenge that questions the efficacy and efficiency of health care delivery systems. Heart failure (HF) remains a leading cause of hospital admissions and subsequent rehospitalizations worldwide.^1^,^2^ HF is a major threat to human wellness and socioeconomic development and continues to rise in prevalence.^3^

The burden is particularly pronounced in low- and middle-income countries (LMICs), especially in South Asia, where HF significantly contributes to morbidity and mortality in young and middle-aged populations.^1^,^4^ Pakistan is no exception, facing systemic challenges such as a shortage of hospital beds, underdeveloped infrastructure, and limited intensive care capacity.^5^ Moreover, the country’s health workforce density falls below the minimum threshold recommended by the World Health Organization (WHO), impairing efforts to achieve universal health coverage and meet health-related Sustainable Development Goals (SDGs), particularly SDG 3.4 aimed at reducing non-communicable diseases (NCD) by one-third by 2030.^6^ Unplanned rehospitalizations further strain healthcare resources and compromise care accessibility and quality.^7^

Among the emerging contributors to adverse HF outcomes, malnutrition and frailty have gained increasing attention. Both are prevalent and share overlapping pathophysiological mechanisms that exacerbate functional decline, hinder recovery, and increase the risk of recurrent hospitalizations.^8^ Malnutrition-induced frailty, marked by progressive physical weakness, exhaustion, and weight loss, is linked to poor HF prognosis.^8^,^9^ Despite this, their combined influence on time-to-event outcomes like rehospitalization remains under-investigated, particularly in South Asia.

While many studies have examined malnutrition or frailty as independent predictors using cross-sectional data or static risk models, survival analysis offers a more robust and dynamic approach.^9^,^10^ It enables not just whether an event occurs, but also when, which is essential in HF management where early rehospitalizations signal deterioration. Prospective studies examining how malnutrition-induced frailty interacts with sociodemographic and clinical variables such as age, comorbidities, body mass index (BMI), and physical activity in influencing the risk and timing of rehospitalization remain limited in LMICs.^7^

A predictive model using logistic regression was previously developed from the same cohort, which identified malnutrition-induced frailty as a significant predictor of rehospitalization in HF patients. However, logistic models are limited in accounting for varying follow-up times and event timing. To address this and complement prior findings, this study employs Kaplan-Meier survival analysis and Cox proportional hazards regression to assess the impact of malnutrition-induced frailty on time to rehospitalization in HF patients.

The study aimed to determine the effect of malnutrition-induced frailty on time to rehospitalization among HF patients estimating survival distributions for exposed and unexposed groups using Kaplan-Meier analysis; and identifying factors associated with time to rehospitalization using Cox regression.

## METHODOLOGY

This prospective cohort study was conducted at the Karachi Institute of Heart Diseases (KIHD) from November 2024 to April 2025. Patients with confirmed heart failure were enrolled during index admission and followed for 90 days to assess weekly time to rehospitalization. Participants were excluded if they had frailty unrelated to malnutrition, poor medication adherence, or comorbidities associated with frailty (e.g., liver or renal disease, malignancy, autoimmune conditions, psychiatric illness, or chronic infections) as verified through clinical records, laboratory data, and medical history.

### Ethical approval:

Ethical approval was obtained from the Institutional Review & Ethics Board (IR&EB) of Baqai Medical University (Ref: BMU-IREB/10-2024/013; dated October 14, 2024) and the Institutional Review Board of KIHD (Ref: IRB/KIHD/Approval/2024/002; dated November 15, 2024). Written informed consent was obtained from all participants and confidentiality was maintained using coded identifiers.

Malnutrition risk was assessed using the Malnutrition Universal Screening Tool (MUST; score ≥1), and frailty using the Tilburg Frailty Indicator (TFI; score >5). Patients meeting both criteria were classified as the frail group (malnutrition and frailty both); while those with neither formed the non-frail group. Patients whose baseline exposure changed at rehospitalization were excluded to preserve group consistency.

Sample size was calculated using an online Cox regression calculator (www.sample-size.net), assuming a hazard ratio (HR) of 1.78,^11^ 95% confidence level, and 80% power. The required sample size was 252, inflated to 300 to account for attrition. Patients were recruited using non-probability consecutive sampling, whereby all eligible participants presenting during the study period was enrolled sequentially until the target of 150 patients in each group was reached. Baseline data were collected through structured interviews and hospital records, including sociodemographic, behavioral, anthropometric, and clinical variables. MUST and TFI scores were recorded at enrollment. The outcome was time to first rehospitalization, predictors included malnutrition, frailty, age, gender, BMI, education, income, smoking status, other addictions (chew tobacco/gutka/niswar), comorbidities and physical activity.

### Statistical analysis:

SPSS 26.0 (IBM Corp., Armonk, NY, USA) was used for data analysis. The Shapiro-Wilk test was applied to assess normality of continuous variables. Normally distributed continuous data were described as means + standard deviations; categorical data as n (%). Independent t-test and Chi-square tests were used to compare groups. Spearman’s correlation was used to assess the relationship between malnutrition and frailty. Kaplan-Meier curves estimated survival, and the log-rank test assessed the association between the predictor and outcome. Cox proportional hazards regression identified independent predictors of rehospitalization, with 95% confidence intervals (CIs). A p-value <0.05 was considered statistically significant.

## RESULTS

Baseline characteristics by rehospitalization status is shown in Table-I. No significant associations were found with sociodemographic or behavioral factors, including smoking and other addiction (substance use) and physical activity, with rehospitalized and not-rehospitalized patients (p>0.05). However, underweight BMI and frailty status were strongly associated with rehospitalization (p<0.001), highlighting the impact of malnutrition and frailty. A strong positive correlation between MUST and TFI scores at enrolment (Spearman’s ρ = 0.889, p < 0.001) confirmed a significant link between malnutrition risk and frailty severity, supporting the exposure classification used in survival analyses.

**Table-I:** Association of Sociodemographic, Behavioural, Lifestyle and Clinical Profile Variables with rehospitalization status (N = 300).

Variable		No Rehospitalization		Rehospitalization		p-Value
Continuous		N=226		N=74		
Age (years)	Mean + SD*	63.24 + 10.39		64.24 + 9.57		0.465
*Categorical*		*n*	*%*	*n*	*%*	
Age Categories (years)	Less than 60	93	41.2	28	37.8	0.876
	61-70	85	37.6	29	39.2	
	71 or more	48	21.2	17	23.0	
Gender	Male	133	58.8	39	52.7	0.417
	Female	93	41.2	35	47.3	
Marital Status	Single	82	36.3	29	39.2	0.679
	Married	144	63.7	45	60.8	
Educational Status	Uneducated	79	35.0	23	31.1	0.574
	Educated	147	65.0	51	68.9	
Employment Status	Unemployed	81	35.8	24	32.4	0.606
	Employed	56	24.8	16	21.6	
	Housewife	89	39.4	34	45.9	
Monthly household Income	Less than 38000	42	18.6	15	20.3	0.440
	38001-50000	81	35.8	21	28.4	
	50001-100000	75	33.2	24	32.4	
	100000 or more	28	12.4	14	18.9	
Smoking Status	Non Smoker	158	69.9	48	64.9	0.624
	Smoker	30	13.3	13	17.6	
	Ex-Smoker	38	16.8	13	17.6	
Other Addiction (Substance Use) (Chew Tobacoo/Gutka/ Niswar)	No	170	75.2	59	79.7	0.529
	Yes	56	24.8	15	20.3	
Physical Activity Status	No	186	82.3	54	73.0	0.094
	Yes	40	17.7	20	27.0	
Frequency of Physical Activity/Week	No Physical Activity	186	82.3	54	73.0	0.115
	1-2 day/week	11	4.9	2	2.7	
	3-5 days/week	22	9.7	14	18.9	
	6-7 days/week	7	3.1	4	5.4	
Time Duration of Physical Activity	No Physical Activity	186	82.3	54	73.0	0.173
	Less than10 minutes	9	4.0	6	8.1	
	More than 10 minutes	31	13.7	13	18.9	
Body Mass Index (At Rehospitalization)	Below 18.5 (Underweight)	9	4.0	26	35.1	<0.001
	18.5-24.9 (Normal Weight)	121	53.5	25	33.8	
	25.0-29.9 (Overweight)	55	24.3	17	23.0	
	30.0-34.9 (Obese)	41	18.1	6	8.1	
Heart Failure Type	Systolic Failure (< 40%)	165	73.0	53	71.6	0.292
	Diastolic Failure(41-49%)	26	11.5	5	6.8	
	Diastolic Failure (> 50%)	35	15.5	16	21.6	
Co-morbid Disease Status	No	90	39.8	22	29.7	0.130
	Yes	136	60.2	52	70.3	
Frailty Status	Non-Frail	134	59.3	16	21.6	<0.001
	Frail	92	40.7	58	78.4	

Table-II compares survival times between frail and non-frail HF patients. The non-frail group had a longer mean time to rehospitalization (9.375 weeks; 95% CI: 8.334-10.416) than the frail group (6.897 weeks; 95% CI: 6.282-7.511). Median survival time followed a similar pattern: 9.0 weeks versus 6.0 weeks, respectively. The log-rank test (χ² = 6.926, df = 1, p = 0.008) indicated a significant difference in survival distributions, with exposure status linked to earlier rehospitalization.

**Table-II:** Survival Analysis: Comparison of Time (in weeks) to rehospitalization in Exposed vs. Unexposed HF Patients.

Patient Exposure Status	Survival Time (in Weeks)							
	Mean				Median			
	Estimate	Std. Error	95% Confidence Interval		Estimate	Std. Error	95% Confidence Interval	
			Lower Bound	Upper Bound			Lower Bound	Upper Bound
Non-Frail	9.375	.531	8.334	10.416	9.000	.667	7.693	10.307
Frail	6.897	.314	6.282	7.511	6.000	.381	5.254	6.746
Overall	7.432	.295	6.854	8.011	7.000	.387	6.241	7.759

**Fig.1:**
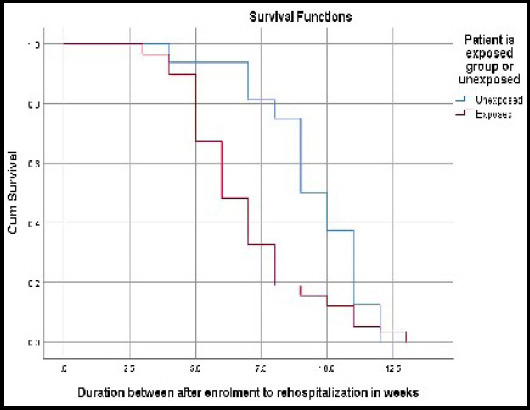
Kaplan Meier Survival Curves for rehospitalization over time (in Weeks) in Exposed vs. Unexposed HF Patients.

Kaplan-Meier survival curves for time to rehospitalization, illustrating a clear separation between frail and non-frail HF cohorts is shown in Fig-1. The frail group showed consistently lower cumulative survival probability over time, with a steeper decline in the survival function indicating a higher and earlier incidence of rehospitalization and increased vulnerability associated with the exposure.

**Fig.2:**
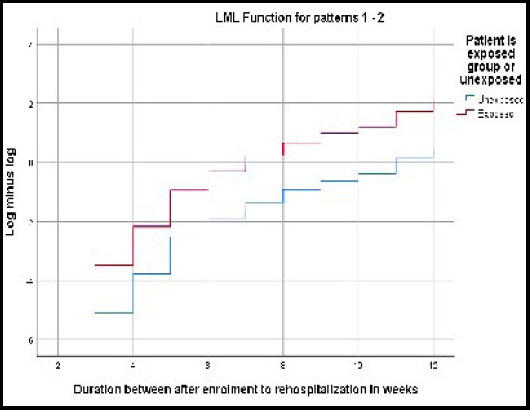
Assessment of Cox Proportional Hazards Model Assumption: Log-Minus-Log Survival Curves of Exposed and Unexposed HF Patients..

A multivariable Cox proportional hazards regression model was applied to assess independent predictors of time to rehospitalization in HF patients, adjusting for key sociodemographic and clinical variables. The exposure variable (frail vs. non-frail group) showed a higher hazard for rehospitalization (HR = 1.763; 95% CI: 0.827-3.761), but this association was not statistically significant (p = 0.142), suggesting exposure alone was not an independent predictor after adjustment. Among the variables, only BMI category at enrollment emerged as a statistically significant predictor (p = 0.047), with underweight patients facing a higher risk of rehospitalization than those in normal, pre-obese, or obese BMI ranges. Frailty, although clinically relevant, was not a statistically significant factor in this adjusted model.

Using the forward Wald method, BMI category at enrollment emerged as a significant predictor in the model, with normal and pre-obese BMI categories showing statistically significant associations with the outcome (Table-III). These findings indicate a potential protective effect of normal, pre-obese and obese BMI on rehospitalisation risk compared to underweight status.

**Table-III:** Cox Regression Model for rehospitalisation (Forward Wald test).

Variables	B	SE	Wald	df	Sig.	Exp(B)	95.0% CI for Exp(B)	
							Lower	Upper
BMI - Underweight			13.885	3	.003			
BMI - Normal Weight	-.964	.296	10.595	1	.001	.381	.213	.681
BMI - Overweight	-1.032	.327	9.957	1	.002	.356	.188	.676
BMI - Obese	-.705	.458	2.369	1	.124	.494	.201	1.213

To explore potential effect modification, an interaction term between frailty and BMI was introduced into the Cox regression model. Upon inclusion, the main effects of frailty and BMI lost statistical significance, and the interaction itself was also not significant (p = 0.233). These findings suggest BMI does not significantly modify the association between frailty and time to rehospitalization in HF patients. While BMI remains an important individual prognostic indicator, its interaction with frailty does not meaningfully affect rehospitalization risk in this cohort.

The log-minus-log (LML) survival plot was used to visually assess the proportional hazards assumption of the Cox model. The curves for exposed (frail) and unexposed (non-frail) patients followed generally parallel paths, indicating that the assumption was reasonably met and supporting the validity of applying the Cox model. The curve for the frail group consistently lay above that of the non-frail group, suggesting a higher hazard of rehospitalization. This trend align with the Cox model estimate (HR = 4.912; 95% CI: 0.752-32.071), although not statistically significant (p = 0.096). Overall, the LML plot supports model assumptions and suggest increased risk of rehospitalization among frail patients.

## DISCUSSION

This prospective study examined the impact of malnutrition-induced-frailty on time to rehospitalization in HF patients in a tertiary care setting. The primary aim was to determine whether frailty due to malnutrition was associated with a shorter duration to rehospitalization. Survival analysis showed that frail patients had significantly shorter rehospitalization-free time than non-frail patients (log-rank p = 0.008), underscoring frailty as a clinically relevant factor.

Kaplan-Meier curves revealed a clear divergence in survival probability between the two groups. Multivariable Cox regression showed that frailty was associated with a higher hazard of rehospitalization, though not statistically significant (p = 0.096) after adjustment. This trend aligns with prior studies linking frailty to adverse HF outcomes.^9^,^12^,^13^ The lack of statistical significance and the wide confidence intervals may reflect limited sample size or misclassification bias therefore, should be interpreted with caution.^14^,^15^ Frailty, a multidimensional vulnerability, exacerbates the physiological stress of HF, increasing susceptibility to adverse outcomes.^16^ Evidence suggest that even non-significant frailty may still carry clinically meaningful prognostic implications.^17^

In the adjusted Cox model, most sociodemographic and behavioral variables showed no significant association with rehospitalization risk, consistent with LMIC studies where structural and clinical factors often exert greater influence on health outcomes.^1^,^18^ In the context of present study conducted in Pakistan, factors such as variability in access to care, discharge planning protocols, and nutritional support may have contributed to these findings.

Among covariates, baseline BMI category emerged as the only statistically significant predictor of time to rehospitalization (p = 0.047). Underweight patients had a higher hazard of rehospitalization than those with normal, pre-obese, or obese BMI. This significant association shows that nutritional status should be considered in conjunction with frailty in HF patients. Although BMI was not the primary exposure of interest, it was included in the model, and its significant association with rehospitalization highlights its clinical importance. This observation is consistent with evidence linking low BMI in HF to adverse outcomes due to reduced nutritional reserves, sarcopenia, and impaired physiological resilience.^9^,^19^ These findings aligns with the “obesity paradox” in HF, where overweight and mildly obese patients may experience better short-term survival and reduced rehospitalisation rates.^20^ However, the interaction analysis showed BMI did not significantly modify frailty’s impact, suggesting both may independently influence outcomes.^21^ This reinforces the need to view malnutrition-induced frailty as a distinct clinical entity that warranting targeted management.

We tested the Cox model assumptions using the Schoenfeld residual-based global test. The results indicated no violation of the proportional hazards assumption (p > 0.05), supporting the validity of the Cox regression model. The proportional hazards assumption was reasonably met, as shown by parallel log-minus-log survival curves. The frail group’s curve consistently lay above that of the non-frail group, indicating higher hazard and supporting the Cox model’s validity to analyze time-to-rehospitalization data.^22^ Clinically, these findings emphasize the importance of routine frailty screening in hospitalized HF patients. Early identification of frailty, independent of BMI could enable timely interventions such as nutritional support and closer post-discharge surveillance.^23^,^24^ In resource-constrained settings, such strategies may reduce preventable rehospitalizations and improve quality of life.^25^

This study contributes region-specific evidence from Pakistan, where reliable data on frailty and rehospitalisation are scarce. Malnutrition, being a major public health issue across South Asia including Pakistan, the findings of the current study emphasises the unmet need to integrate screening assessment for nutrition and frailty into HF care in developing countries. Moreover, the prospective study design, strict inclusion and exclusion criteria and the use of standardised universally validated scales further strengthen the validity of the findings and highlight their relevance to clinical practice in LMICs.

### Limitations

The relatively small sample size and short follow-up may have limited statistical power. Despite controlling confounders, such as poor medication adherence and comorbidities, some unmeasured variables including psychosocial support and dietary diversity, were not assessed. The absence of randomization may also have affected cohort representativeness and limit the generalisability of the results. However, due to the limited resources and practical constraints of hospital setting, consecutive sampling was the most feasible method for timely recruitment. The findings are clinically relevant however, the lack of statistically significant associations limits the strength of inference. Even with these constraints, the study provides meaningful insights and strong evidence from LMICs context, highlighting the importance of incorporating frailty-focused strategies into HF management.

## CONCLUSION

This study highlights the clinical relevance of malnutrition-induced frailty in predicting time to rehospitalization among HF patients. Malnutrition-Induced frailty showed a shorter a shorter survival time to rehospitalisation in HF patients and a trend towards increased hazards, though this association was not statistically significant. Various influencing factors such as psychosocial conditions and dietary diversity might also influence rehospitalisation. However, the findings support the integration of routine frailty screening and nutritional assessment into standard HF management, particularly in resource-limited settings.

### Recommendations

Future multicenter research with larger sample and more diverse South Asian cohorts, longer follow-up and incorporation of unmeasured variables should further validate these findings.
